# Maximal inspiratory and expiratory flow at moderate altitude: a study of a Latin American population

**DOI:** 10.1186/s12890-022-01943-x

**Published:** 2022-04-19

**Authors:** Laura Gochicoa-Rangel, Keylin Yaoska Rodríguez-Peralta, Ana Karen Gutiérrez-Bautista, Carlos Guzmán-Valderrábano, Rosario Fernández-Plata, Luis Torre-Bouscoulet, David Martínez-Briseño

**Affiliations:** 1 Department of Respiratory Physiology, National Institute of Respiratory Diseases “Ismael Cosío Villegas”, Mexico City, Mexico; 2 Department of Hospital Epidemiology and Infectology, National Institute of Respiratory Diseases “Ismael Cosío Villegas”, Tlalpan 4502, Section XVI, Mexico City, 14080 Mexico; 3 Pulmonary Function Laboratory, Institute for Development and Innovation in Respiratory Physiology S de RL, Mexico City, Mexico

**Keywords:** Peak inspiratory flow, Peak expiratory flow, Moderate altitude, Reference values, Spirometry, Flowmeter

## Abstract

**Background:**

Peak inspiratory and expiratory flows (PIF, PEF) are parameters used to evaluate the mechanics of the respiratory system. These parameters can vary based on whether they are measured using mechanical devices vs. spirometry and based on the barometric pressure at which the measurements are obtained. Our objectives were (1) to report the normal values and variability of PEF and PIF of a Latin American population living at a moderate altitude (2240 m above sea level), (2) to analyze the adjustment of reference values obtained at sea level with those obtained in healthy subjects living at a moderate altitude, and (3) to assess the correlation between PEF obtained by spirometry (PEFs) and PEF obtained by mechanical devices (PEFm).

**Methods:**

In this prospective and transversal study, men and women with good respiratory health aged between 2.8 and 68 years old were invited to participate. Randomly, they underwent spirometry (to measure PEFs and PIFs) and mechanical flowmetry (to measure PEFm).

**Results:**

A total of 314 subjects participated, with an average age of 24.3 ± 16.4 years; 59% were Women. The main determinants for the reference equations were age, weight, height and sex at birth. The agreement of the PEFm, PEFs and PIFs values was inconsistent with that reported by other authors, even at the same barometric pressure. The association between PEFm and PEFs was r = 0.91 (p < 0.001), and the correlation coefficient of concordance was 0.84.

**Conclusions:**

The PEFm, PEFs, and PIFs measurements in individuals living at moderate altitudes are different from those found by other authors in cities with different barometric pressures and ethnicities.

**Supplementary Information:**

The online version contains supplementary material available at 10.1186/s12890-022-01943-x.

## Background

Peak inspiratory and expiratory flows (PIF and PEF, respectively) are effort-dependent physiological parameters that are used to assess the mechanics of the respiratory system and provide information on muscle strength, airway caliber, and lung elastic characteristics [1, 2]. PIF/PEF measurements can be performed with a spirometer or by mechanical devices called flowmeters [3].

Mechanical flowmeters for PEF measurement (PEFm) are widely available and have been used primarily in subjects with asthma for home monitoring purposes [4]. These devices help to evaluate airflow limitation and airway response to bronchodilators, allowing the timely detection of decreased expiratory flow [5, 6]. PIF is mainly measured by spirometry (PIFs) and can be very useful in investigating abnormalities in the distal and proximal airways and for outpatient follow-up during pulmonary rehabilitation [7, 8]. Mechanical flowmeters for PIF have been used to determine the most appropriate inhalation device in the treatment of asthma and chronic obstructive pulmonary disease (COPD) [9].

Compared to PEF measurements, PIF measurements are less useful due to lack of available low-cost equipment and reference equations [10, 11] in addition to high test variability and limited knowledge of its clinical applications [12, 13].

Most of the reference equations for PEFm are based on European and Asian populations living at sea level, and although some studies reported a good correlation between PEFm and those obtained by spirometry (PEFs), some authors have reported that PEFm may underestimate the value of expiratory flow when obtained in cities that are at elevations above sea level, probably due to air density [14, 15]. On the other hand, very few studies have reported normal PIF values [10, 11].

The aims of the present study were (1) to report the normal values and variability of PEFm, PEFs and PIFs for a Latin American population living at a moderate altitude; (2) to analyze the adjustment of reference values obtained at sea level with values obtained in healthy subjects living at a moderate altitude; and (3) to assess the correlation between PEFm and PEFs.

## Material and methods

### Study design and population

A cross-sectional study was conducted at the Department of Respiratory Physiology of the National Institute of Respiratory Diseases in Mexico City between April and September 2019. Healthy men and women, aged 2.8–68.0 years without cardiovascular, hepatic or renal diseases or their antecedents of prematurity, pneumonia, bronchiolitis, or biomass tobacco smoke exposure were invited to participate. These individuals were recruited from schools and workplaces. Those who agreed to participate were required to sign an informed consent form. The study was approved by the Ethics Committee of the institute with the number C12-19.

### Anthropometric and pulmonary measurements

The height (cm) and weight (kg) of all the participants were measured using a stadiometer with a scale (Models 206 and 769, Seca, Hamburg, Germany). In addition, participants underwent spirometry, and flowmetry was randomly assigned.

To obtain the PEFs and PIFs, an EasyOne On-PC spirometer (NDD, Zurich, Switzerland) was used. After verification of the spirometer calibration, the maneuver was performed according to American Thoracic Society (ATS) and European Respiratory Society (ERS) 2005 standards [16]. While the participant was sitting upright and with a nasal clip, he or she was asked to breathe at a tidal volume, to perform maximum inhalation and to blow hard and continuously through a sterile mouthpiece until the criterion of forced exhalation was met, and then, the participant was asked to perform a fast and maximum inhalation [16]. The maneuver was repeated until the acceptability and repeatability criteria of the test were met (abrupt and vertical onset, with peak expiratory flow, triangular flow-volume curve, with an extrapolated volume ≤ 150 mL). A 6-s exhalation (or 3 s in children) with a plateau at the end of the forced exhalation of at least 1 s and free of artifacts), the highest value of PEFs and PIFs from the 3 best efforts were used for the analysis.

The PEFm was performed with a Truzone® device (peak flow meter, Monaghan Medical 96510, New York, USA); likewise, with the participant sitting with their back upright and their head slightly raised, they were asked to take a quick, deep breath in and immediately afterward to seal their lips around the mouthpiece and perform a quick, strong exhalation. The PEFm maneuver was repeated 3 times, and the highest value obtained was used for analyses [12].

### Statistical analysis

The general characteristics of the population are expressed as the median (minimum–maximum) value.

The coefficient of variation (CV, [standard deviation/mean]*100) among the three measurements of PIFs, PEFs and PEFm was calculated for each participant to estimate within-subject variability. Box plots were used to report the median (p25, p75) coefficient of variation for the sex and age groups (< 10, 10–20, 20–40, > 40).

For the agreement and association analysis, the unit of the PEFm variable was transformed to liters/second (L/s) to be the same as the value obtained by spirometry; for the calculation of the regressions and graphs, the PEFm was kept in its original unit liters/minute (L/min). To evaluate the degree of agreement, the mean of differences, and the 95% limits of agreement between PEFm and PEFs measurements, the concordance correlation coefficient (CCC) and Bland–Altman plot were used. The variables did not follow a normal distribution; therefore, the associations between dependent variables (PEFm, PEFs and PIFs) and independent variables (age, weight, height) were calculated using the Spearman’s correlation coefficient (r).

In regression models, the dependent variables were transformed using the natural logarithm, while the independent variables remained at their original value. Multiple linear and quantile regression were fitted to estimate the reference equations for the mean and the lower limit of normal (LLN, defined as the 5^th^ percentile), respectively. The coefficient of determination (R^2^, linear model) and pseudo coefficient of determination (pseudo-R^2^, quantile model) were reported to describe the percentage of variation explained in spirometric variables by the independent variables. Root mean square error (RMSE) was reported as a measurement of goodness of fit.

The obtained values were compared with reference equations from other authors [10, 11, 14, 17–28]. Results with a p < 0.05 value was considered statistically significant.

The data were collected using Microsoft Excel version 16.39. Statistical analysis was performed with STATA software (version 12; StataCorp, College Station, TX).

## Results

**Fig. 1:**
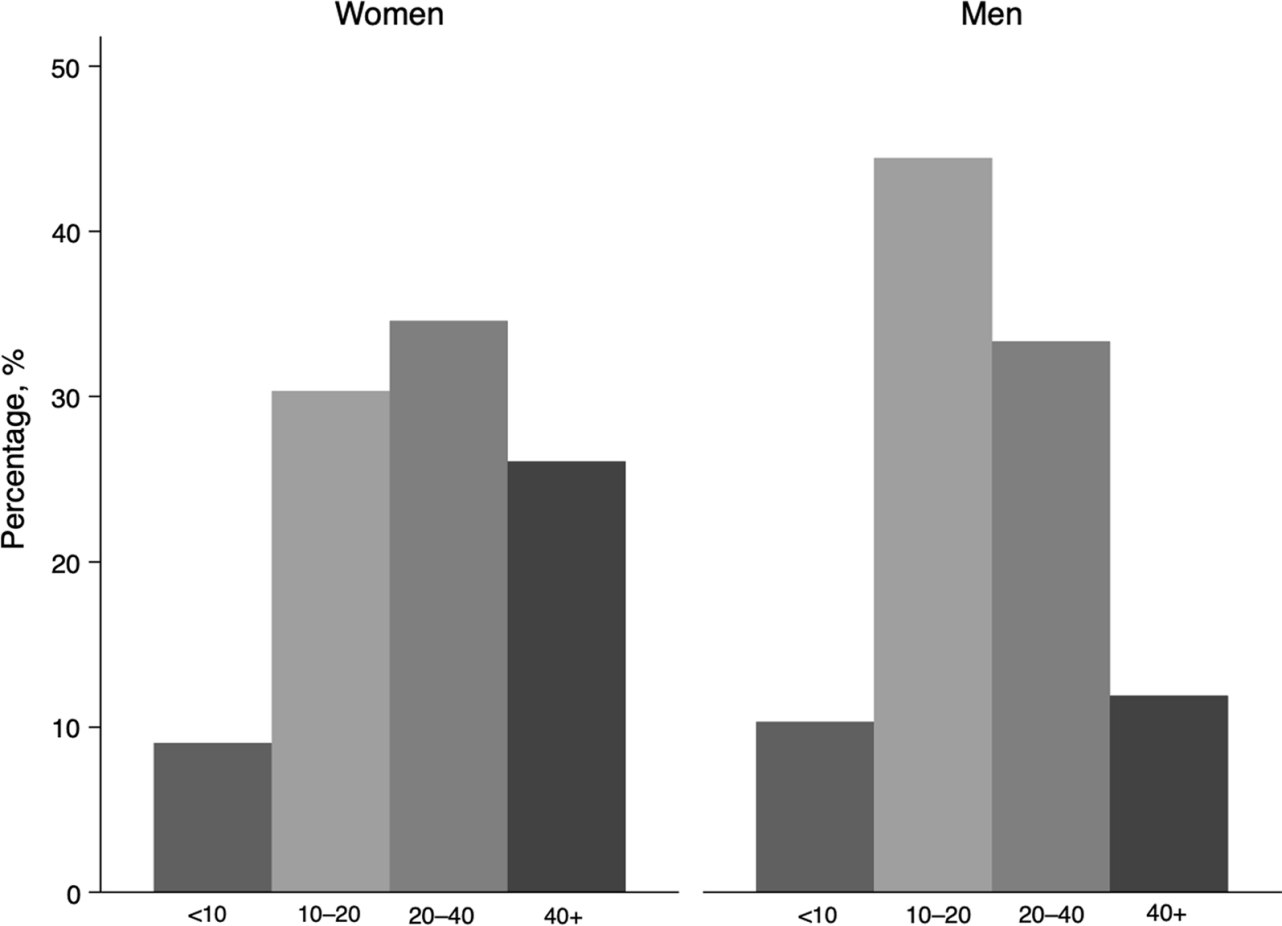
Histogram showing the age of the participants according to age groups and sex (n = 314)

A total of 314 participants were recruited, 188 (59.9%) of whom were Women. The entire group had a median age of 21.8 years (2.8–68.0). Table 1 describes the anthropometric and spirometric characteristics of the population. Figure 1 describes the distribution of the sample according to sex and age groups.

**Table 1:** General characteristics of the participants

Variables	All, n = 314 Median (min -max)	Women, n = 188 Median (min -max)	Men, n = 126 Median (min -max)
Age, years	21.8 (2.8–68.0)	24.5 (2.8–68.0)	14.0 (4.2–61.0)
Weight, kg	58 (13–124)	58 (13–115)	56 (17–124)
Height, cm	155 (92–186)	155 (92–174)	159 (105–186)
PEFm, L/m	360 (70–810)	360 (70–670)	400 (90–810)
PEFs, L/s	7.1 (1.2–14.6)	6.9 (1.2–10.9)	7.7 (1.3–14.6)
PIFs, L/s	4.6 (0.7–13.3)	4.6 (0.9–10.9)	4.7 (0.7–13.3)

PEFm, peak expiratory flow measured by mechanical flowmeter; PEFs, peak expiratory flow measured by spirometry; PIFs, peak inspiratory flow measured by spirometry

**Fig. 2:**
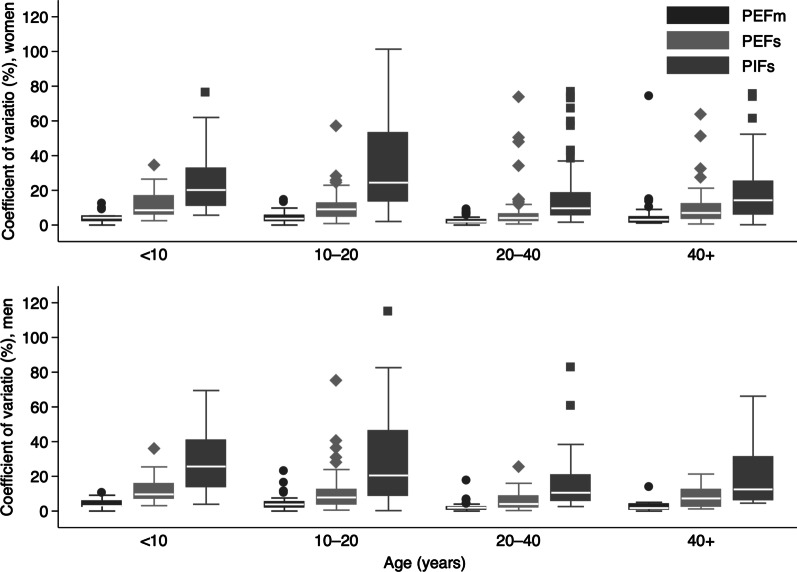
Box plot of coefficient of variation for PEFm, PEFs and PIFs grouped by sex and age (n = 314)

Figure 2 is a box plot for CV according to sex and age group for PEFm, PEFs and PIFs. Variables measured by spirometer had the highest variability regardless of sex and age. Medians of CV for PEFm were under 5% for both women and men and for all the age groups.

**Fig. 3:**
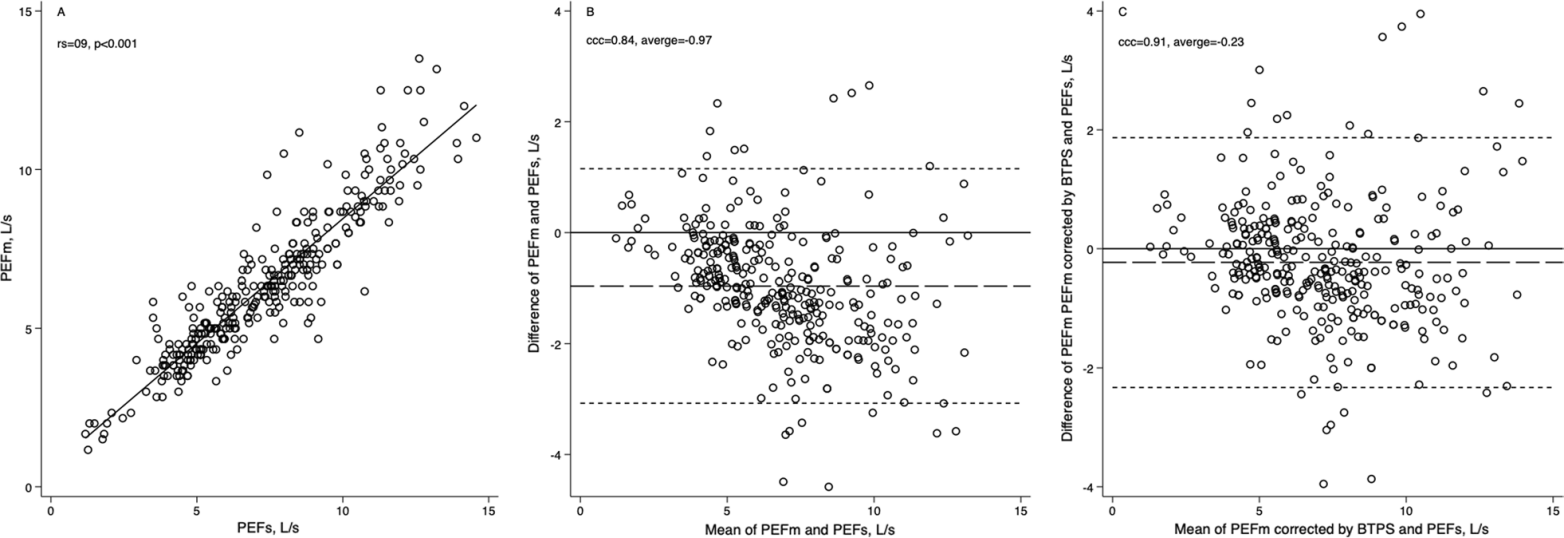
**A** Spearman correlation coefficient and **B** Bland–Altman for peak expiratory flow measured with the flowmeter (PEFm) and spirometer (PEFs) in healthy subjects. (n = 314). The short-dash line represents the mean of the PEFm and PEFs, and the long-dash lines represent the 95% limits of agreement

The correlation between PEFm and PEFs was 0.91 (p < 0.001) (Fig. 3A). The CCC was 0.84, and the mean difference was − 0.97 ± 1.1 L/s with 95% limits of agreement from − 3.07 to 1.15 L/s. Figure 3B depicts the Bland and Altman graph, which describes that PEFm measurement tends to underestimate the PEFs. Adjusted by body temperature, pressure, and water vapor saturated (BTPS), Fig. 3C shows that the mean differences between PEFs and PEFm decreased to − 0.23 ± 1.1 L/s as well as the 95% limits of agreement (from − 2.32 to 1.87 L/s) with a CCC equal to 0.91.

Table 2 reports the Spearman’s correlation coefficients between spirometric and independent variables. Age, height and weight were statistically significant for men and women. In a multivariate analysis, the best models were those in which the variables of PEFm, PEFs and PIFs were transformed into natural logarithms, while the independent variables remained in their original units.

**Table 2:** Spearman’s correlation coefficient between PEFm, PEFs and PIFs and independent variables (n = 314)

Variable	Women (n = 188)			Men (n = 126)		
	PEFm	PEFs	PIFs	PEFm	PEFs	PIFs
Age, years	0.60	0.56	0.49	0.87	0.85	0.71
Height, cm	0.70	0.76	0.63	0.85	0.83	0.75
Weight, Kg	0.65	0.65	0.59	0.85	0.85	0.78

PEFm, PEFs and PIFs were transformed to logn. All associations had a p value < 0.01

In Table 3, regression models for the mean and the lower limit of normality (LLN) for PEFm, PEFs and PIFs are described.

**Table 3:** Regression models for the mean and the lower limit of normality for PEFm, PEFs and PIFs (n = 314)

Variable	Ln(PEFm)		Ln(PEFs)		Ln(PIFs)	
	Mean	LLN	Mean	LLN	Mean	LLN
Sex	0.11748^*^	0.17012^*^	0.08871^†^	0.07958	0.04547	− 0.06546
Weight, kg	0.00312^*^	0.00035	0.00275^†^	0.00232	0.00647^†^	0.00888
Height, cm	0.01175^*^	0.01258^†^	0.01262^†^	0.01564^*^	0.01340^†^	0.01458^*^
Age, years	0.03295^*^	0.05106^†^	0.04317^†^	0.05954^†^	0.03818^†^	0.01657
Age^2^, years	− 0.00042^*^	− 0.00069^†^	− 0.00057^†^	− 0.00083^†^	− 0.00053^†^	− 0.00024
Constant	3.37938^*^	2.89981^†^	− 0.80658^†^	− 1.70366^*^	− 1.44864^†^	− 2.01063
RMSE	0.16548	0.3334	0.16946	0.3439	0.30778	0.6486
R^2^	0.82		0.83		0.65	
Pseudo-R^2^		0.62		0.65		0.46

*p-value < 0.05

^†^p-value < 0.01

R^2^, Coefficient of determination; Pseudo-R^2^, pseudo coefficient of determination; RMSE, Root Mean Square Error; LLN, lower limit of normality; PEFm, PEFs and PIFs were transformed to log_n_. All associations had a p < 0.01. PEFm, peak expiratory flow measured with mechanical device; PEFs, peak expiratory flow measured by spirometry; PIFs, peak inspiratory flow measured by spirometry

**Fig. 4:**
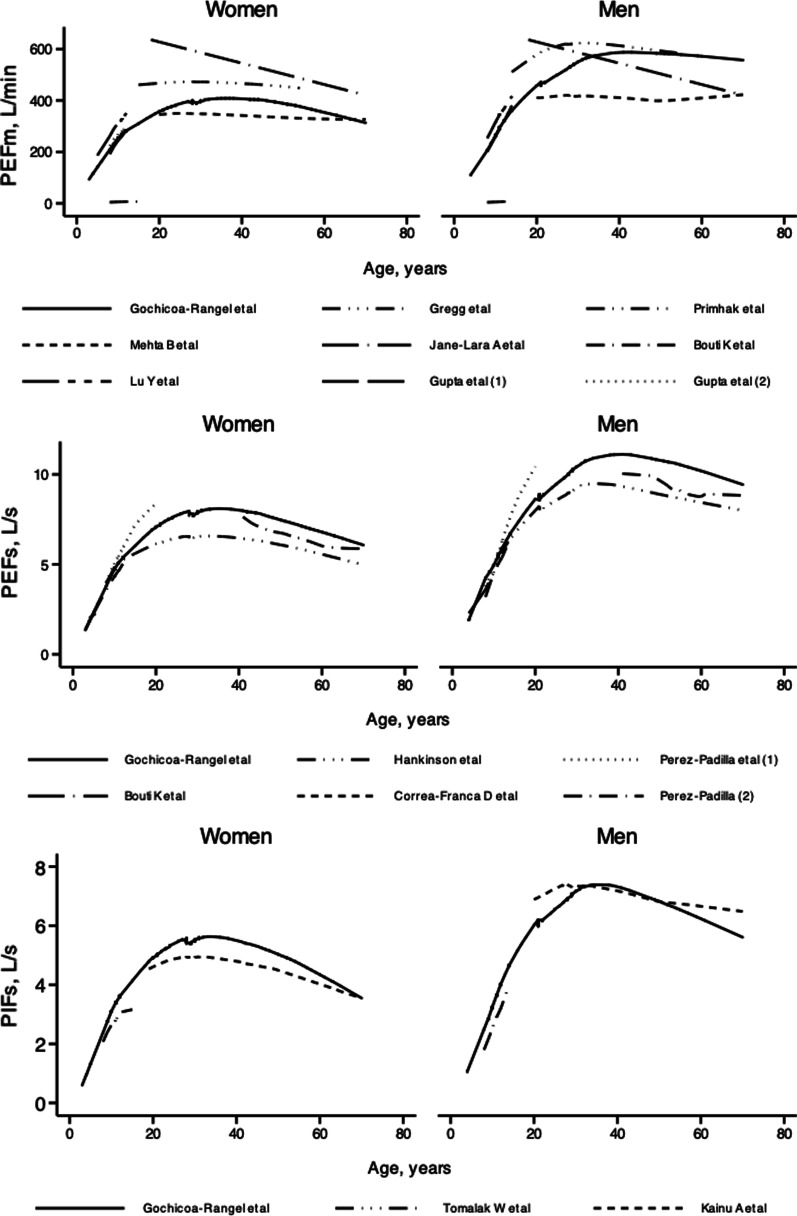
Regression lines (lowess) for **A** peak expiratory flow measured with the flowmeter, **B** spirometer and the **C** peak inspiratory flow measured by spirometry for women and men obtained in the present study and those already reported by several authors (stratifying by sex). PEFm, peak expiratory flow measured with a flowmeter; PEFs, peak expiratory flow measured with a spirometer; PIFs, peak inspiratory flow measured with a spirometer

The results obtained from PEFm, PEFs and PIFs were compared with those reported by other authors (Additional file 1: Table S1). Table 4 shows the results of the concordance analysis, and Fig. 4 shows these same comparisons between the different authors.

**Table 4:** Agreement between the results obtained in the study and published reference values

Reference values	Age (years)	CCC	Average differences* ± SD	95% limits of agreement	r
PEFm (L/min)					
Gregg I. (UK, 35 masl)	14–54	0.55	− 47.9 ± 79	(− 202.9, 106.9)	0.70
Primhak RA. (UK, 33–200 masl)	7–16	0.33	− 68.3 ± 46.9	(− 160.0, 23.8)	0.60
Mehta B.. (India, 231 masl)	7–15	0.53	− 20.8 ± 42	(− 104.5, 62.8)	0.63
Gupta S. (India, 2150 masl)	7–14	0.61	4.4 ± 38.9	(− 71.9, 80.8)	0.66
Gupta S. (India, sea level)	7–4	0.61	− 4.5 ± 39.2	(− 81.5, 72.4)	0.65
Jané-Lara. (Cuba, 59 masl)	18–75	0.09	− 97.7 ± 119.4	(− 331.8, 136.4)	0.17
Lu Y. (China, 21–405 masl)	5–14	0.42	− 51.3 ± 40.3	(− 130.3, 27.7)	0.70
Bouti K. (Marrakech, 24 masl)	3–13	0.80	− 14.4 ± 42	(− 96.7, 67.9)	0.80
Bouti K. (Morocco, 90 masl)	18–70	0.81	4.26 ± 41.5	(− 77.01, 85.6)	0.81
PEFs (L/s)					
Hankinson JL, (USA, NHANES III)	8–80	0.74	− 0.92 ± 1.34	(− 1.7, 3.5)	0.86
Pérez-Padilla, (México, 2240 masl)	8–20	0.66	− 0.33 ± 0.96	(− 2.2, 1.5)	0.68
Pérez Padilla, (Latinamerica)	40–90	0.59	− 0.98 ± 1.5	(− 1.9, 3.8)	0.78
Bouti K, (Marrakech, 24 masl)	3–13	0.68	0.3 ± 0.82	(− 1.3, 1.9)	0.77
Bouti K, (Marrakech, 90 masl)	18–70	0.69	− 0.40 ± 0.83	(− 2.0–1.2)	0.79
Corrêa-Franca, (Brazil, 776 masl)	4–16	0.09	− 0.19 ± 0.45	(− 1.1, 0.7)	0.24
PIFs (L/s)					
Tomalak W, (Poland, 560 masl)	7–15	0.23	0.71 ± 1.04	(− 1.3, 2.78)	0.49
Kainu, (Finaland, 4–560 masl))	19–83	0.43	0.72 ± 1.8	(− 2.8, 4.3)	0.53

*In order to compute the average difference, we used the following procedure: 1. We estimate the difference between PEF or PIF from reference equation–PEF or PIF from this study 2. Finally, the average of the differences was calculated

CCC, concordance correlation coefficient; SD, standard deviation; r, correlation coefficient; PEFm, peak expiratory flow measured by mechanical flowmeter; PEFs, peak expiratory flow measured by spirometry; PIFs, peak inspiratory flow measured by spirometry; masl, meters above sea level

## Discussion

The results of this study confirm that the main determinants for obtaining the reference values of PEFm, PEFs and PIFs were age, weight, height and sex at birth and that the measurements in individuals living at a moderate altitude are not interchangeable with those obtained at sea level.

It is well recognized that mechanical "flowmeters" are useful for monitoring patients with obstructive diseases, mainly for patients with asthma [3, 4, 6, 17], due to their availability, portability, and low cost and that do not require specialized training. PEF measurement, either by spirometry or flowmeters, is used to determine the severity of an asthma attack and to monitor the patient's disease at home [4, 29, 30] and has even proposed its use for the diagnosis of the disease [6, 31, 32].

Although the PEFm and the PEFs have a good correlation, there is not an acceptable agreement between the measurements [14, 20, 21]. Differences can even exist between the measurements obtained from the same subject using the same spirometer. When the measurement is obtained with a maneuver that provides a peak flow measurement only vs. a "complete" spirometry maneuver, the PEF value obtained from a short exhalation tends to be higher than the PEF value obtained while performing a forced vital capacity maneuver (29). In the present study, similar results were obtained, with a high correlation (r = 0.91) but with regular agreement (CCC = 0.84), with errors of up to 3 L/s between the measurements with PEFm vs. PEFs. These differences can be expected since the technology used by both devices is different and because the results of PEFs are adjusted based on the correction factor of BTPS units; however, as we demonstrated in the results, even when we adjust the values of PEFm to BTPS units, the agreement between PEFs and PEFm remains practically the same.

Some authors have made reference equations for PEFm adjusting for the BTPS conversion factor [22]; however, we consider that the predicted values should not be adjusted in the day-to-day use of PEFm to facilitate implementation for patients at home, as well as those in primary care settings.

Another important factor that affects the PEFm measurements is the barometric pressure of the location where the measurement is performed [33, 34]. Studies conducted through simulations of people ascending to a high altitude have shown that contrary to the measurements obtained by spirometry [33], the PEFm value is lower at higher altitudes due to the air density. An adjusted PEFm measurement of a 6.6% per 100 mmHg decrease from 760 mmHg in the barometric pressure has been proposed [15, 34]. Gupta et al. found that children living at 2150 m above sea level (masl) had a higher PEFm than those living at 278 (masl) using a Mini Wright® flowmeter and adjusted the result of the measurements of this flowmeter to BTPS units, including air density [22]. It is possible that this finding is substantial because a higher lung volume is usually expected in individuals living at higher altitudes, and by adjusting for air density, higher values are possible. However, we consider that the usefulness of the PEFm is mainly for individual use and perhaps in the clinical context at the first level of care, so making these adjustments in the reference equations could make it difficult to apply on a day-to-day basis.

As shown in Table 4, the results do not agree with those of other authors; Pérez-Padilla et al. found, by studying children under 20 years of age [25] in a Hispanic population living at 2240 masl by means of spirometry, a stronger association and agreement. Therefore, the results obtained in the present study indicate that PEFm can be obtained in individuals who live at a moderate altitude and are between 3 and 68 years of age, which will improve diagnostic evaluations and patient follow-up.

The reference values used to calculate the PIFs value will be useful in the clinical context to evaluate whether asthma patients with different degrees of control or those who experience asthma attacks could perform the inspiratory flow necessary to inhale the medication of the different devices [35]. PIFs also help in the diagnosis of upper airway diseases [36, 37], in the follow-up of neuromuscular disorders as an indicator of dysphagia or cough effectiveness [38], in the follow-up and management of patients with distal airway obstruction [9, 39], and for the complementary prognostic evaluation in tracheostomy [36]. Currently, to the best of our knowledge, the mechanical devices currently on the market for PIF measurements are designed to control the inspiratory flow that a patient with asthma could generate when inhaling a medication, but this does not provide the PIF of an individual because it has a maximum "stop" measurement [40].

There are few reference values for PIFs in the literature, and as shown in Table 4, similar to PEFm and PEFs values, the PIFs values show poor agreement with values obtained in other populations.

This study has several limitations. The number of participants above 55 years is small; moreover, individuals above 68 years were not included, so the proposed reference equations are limited to these ages. The variability of the PIFs maneuvers was very high, perhaps because we could not follow a standardized method, and this problem is also because of a lack in the literature about the “inspiratory measurements” acceptability and repeatability criteria.

## Conclusions

The measurements of expiratory and inspiratory flows in individuals living at a moderate altitude are different from those found by other authors in cities with different barometric pressures and different ethnicities. Similar to other respiratory function tests, the main determinants of PEFm, PEFs and PIFs values are age, height, weight and sex. We consider that these reference values can be used in the clinical setting for monitoring and diagnosing respiratory diseases in population living at a moderate altitude.

## Supplementary Information

**Additional file 1.** Supplementary material.

## Data Availability

The datasets used and/or analysed during the current study are available from the corresponding author on reasonable request.

## Abbreviations

AIC: Akaike information criterion
COPD: Chronic Obstructive Pulmonary Disease
CV: Coefficient of variation
CCC: Concordance correlation coefficient
PEFm: Mechanical expiratory flow
Masl: Meters above sea level
PEF: Peak expiratory flow
PIF: Peak inspiratory flow
PIFs: Peak inspiratory flow obtained by spirometry
PEFs: Peak expiratory flow obtained by spirometry
SD: Standard deviation
r: Sperman correlation coefficient
