# Supplementary material for: Maximal inspiratory and expiratory flow at moderate altitude: a study of a Latin American population
Source: BMC Pulm Med. 2022 Apr 19;22:147. doi: 10.1186/s12890-022-01943-x (PMC9020005; doi:10.1186/s12890-022-01943-x)
Supplement: Supplementary file 1 — Additional file 1. Supplementary material. [file 12890_2022_1943_MOESM1_ESM.docx]

**Supplementary material**

**Maximal Inspiratory and Expiratory Flow at Moderate Altitude: A study of a Latin American population**

**Reference equations**

Table S1 describes the worldwide reference equations published by PEFm, PEFs and PIFs

Table S1. Worldwide reference equations published by PEFm, PEFs and PIFs

| **Reference values** | **Age (years)** | **Number of participants** | **Reference equation** |
| --- | --- | --- | --- |
| **PEFm (L/min)** |  |  |  |
| Gregg I, 1973 (UK, 35 masl) | 14 - 54 | 401 | Male (14 - 54 years) = -30.15+(30.63*age)-(0.723*age^2)+(0.00521*age^3)+(1.46*height))/60 Female (14-64 years) = 198.07+(3.07*age)-(0.0477*age^2)+(1.42*height))/60 |
| Primhak RA, 1984. (UK, 33-200 masl) | 7 - 16 | 339 | All (7-16 years) = (5.640*height)-472.5)/60 * The result must be multiplied by 60 to obtain Liters/min |
| Mehta B, 2016. (India, 231 masl) | 7 - 15 | 100 | Female (7-15 y) = -277.01+(3.92*height))/60  Male (7-15 y) = -284.55+(4.08*alturacm))/60 |
| Gupta S, 2013. (India, 2150 masl) | 7 -14 | 290 | Male (7-14 y) = -201.25 + (9.25*age) + (2.26*height) + (1.32*weight)/60 Female (7-14 y) = -222.61 + (9.42*edad) + (2.57*alturacm) + (0.11*peso)/60 |
| Gupta S, 2013. (India, sea level) | 7 -14 | 280 | Male (7-14 y) = -150.38 + (9.9*age) + (1.75*height) + (1.74*weight))/60 Female (7-14 y) = -177.06 + (0.86*age) + (2.59*height) + (1.49*weight)/60 |
| Jané-Lara, 2014. (Cuba, 59 masl) | 18 - 75 | 85 | All (18-75 y) =( -0.069*age)+11.84 * The result must be multiplied by 60 to obtain Liters/min |
| Lu Y,2018. (China, 21-405 masl) | 5 -14 | 3,169 | Male (5-14 y) = (4.39*height)-300.48)/60  Female (5-14 y) = ((4.13*heightm)-278.04)/60 |
| Bouti K, 2017. (Marrakech, 24 masl) | 3 - 13 | 222 | Male (3-13 y) = -187+(24.4*age)+(1.61*height))/60 Female (3-13 y) = -151+(17*age)+(1.59*height))/60 |
| Bouti K, 2017. (Morocco, 90 masl) | 18 - 70 | 313 | Male (18 – 67 y) = -374 – (1.63*age) + (5.20*height)/60  Female (18 – 70 y) = -235 – (0.24*age) + (3.48*height)/60 |
| **PEFs (L/s)** |  |  |  |
| Hankinson JL, 199., (USA, NHANES III) | 8 - 80 | 7,429 | Male (<=20 y) =-0.9537-(0.19602*age)+(0.014497*age^2)+(0.00030243*height^2)  Male (>20 y) = 0.0870+(0.06580*age)-(0.001195*age^2)+(0.00030243*height^2) Female (<=18 y) = -3.2549+(0.47495*age)-(0.013193*age^2)+(0.00022203*height^2)  Female (>18 y) =0.2401+(0.06174*age)-(0.001023*age^2)+(0.00022203*height^2) |
| Pérez-Padilla, 2003. (México, 2240 masl) | 8 - 20 | 4009 | Female (<=20 y) = exp(-0.80 +(0.01463*height)+(0.0022*weight)+(0.0218*age))  Male (<=20 y) = exp(-0.536+(0.01190*height)+(0.00236*weight)+(0.0366*age)) |
| Pérez Padilla,2006. (Latinamerica) | 40 - 90 | 906 | Female (40-90 y) =(-0.05360108*age) + (0.0563161*height) + 0.65195532 Male (40-90) =(-0.07185995*age) + (0.06294803*height) + 2.4914045 |
| Bouti K, 2017 (Marrakech, 24 masl) | 3 - 13 | 222 | Male (3-13 y) = -199 + (9.8*age) + (2.67*height)/60 Female (3-13 y) = -181 + (8.5*age)+ (2.5*height)/60 |
| Bouti K, 2017, (Morocco, 90 masl) | 18 - 70 | 313 | Male (18 – 67 y) = -50 – (2.11*age) + (2.61*height) + (2.75*weight)/60  Female (18 – 70 y) = -241 – (0.61*age) + (3.79*height)/60 |
| Corrêa-Franca, 2016. (Brazil, 776 masl ) | 4 - 16 | 195 | All (4-6 y) =exp(-2.908+(0.019 * height)) |
| **PIFs (L/s)** |  |  |  |
| Tomalak W, 2004. (Poland, 560 masl) | 7 - 15 | 296 | Male (7-15 y) = exp(-13.93)* (height^3.003)  Female (7-15 y) = exp(-11.91)* (height^2.593) |
| Kainu, 2018. (Finaland, 4-560 masl)) | 19 - 83 | 368 | All (19-83)= (0.072*height)+(logage*3.719)-(0.107*age)+(1.5*sex)-15.892 *sex= 1(Male) 0 (Female) |
